# A CD4^+^ T cell antagonist epitope down-regulates activating signaling proteins, up-regulates inhibitory signaling proteins and abrogates HIV-specific T cell function

**DOI:** 10.1186/1742-4690-11-57

**Published:** 2014-07-05

**Authors:** Evan S Jacobs, Desmond Persad, Longsi Ran, Ali Danesh, John W Heitman, Xutao Deng, Mark J Cameron, David J Kelvin, Philip J Norris

**Affiliations:** 1 Blood Systems Research Institute, San Francisco, California; 2 Toronto General Research Institute, University of Toronto, Toronto, Canada; 3 University Health Network Vaccine and Influenza Research and Development Center, Toronto, Canada; 4 Division of Immunology, International Institute of Infection and Immunity, Shantou University Medical College, Shantou, People’s Republic of China; 5 Guangdong Provincial Key Laboratory of Infectious Diseases and Molecular Immunolopathology, Shantou, Guangdong, People’s Republic of China; 6 Departments of Laboratory Medicine, San Francisco, California; 7 Medicine, University of California, San Francisco, California

**Keywords:** TCR, Cell signaling, Peptide antagonism, HIV

## Abstract

**Background:**

CD4^+^ T cells are critically important in HIV infection, being both the primary cells infected by HIV and likely playing a direct or indirect role in helping control virus replication. Key areas of interest in HIV vaccine research are mechanisms of viral escape from the immune response. Interestingly, in HIV infection it has been shown that peptide sequence variation can reduce CD4^+^ T cell responses to the virus, and small changes to peptide sequences can transform agonist peptides into antagonist peptides.

**Results:**

We describe, at a molecular level, the consequences of antagonism of HIV p24-specific CD4^+^ T cells. Antagonist peptide exposure in the presence of agonist peptide caused a global suppression of agonist-induced gene expression and signaling molecule phosphorylation. In addition to down-regulation of factors associated with T cell activation, a smaller subset of genes associated with negative regulation of cell activation was up-regulated, including KFL-2, SOCS-1, and SPDEY9P. Finally, antagonist peptide in the absence of agonist peptide also delivered a negative signal to T cells.

**Conclusions:**

Small changes in p24-specific peptides can result in T cell antagonism and reductions of both T cell receptor signaling and activation. These changes are at least in part mediated by a dominant negative signal delivered by antagonist peptide, as evidenced by up-regulation of negative regulatory genes in the presence of agonist plus antagonist stimulation. Antagonism can have dramatic effects on CD4^+^ T cell function and presents a potential obstacle to HIV vaccine development.

## Background

CD4^+^ T cells are critically important in HIV infection as they are the cells that are primarily targeted by HIV and as well play an important role in the immune response to HIV infection [1]. In HIV infection it has been demonstrated that peptide variation can reduce the CD4^+^ T cell response to the virus [2-4]. Peptides can be grouped into three different categories, peptide sequences that elicit full activation phenotypes (agonist sequences), partial activation phenotypes (partial agonists) [5], and others that inhibit CD4^+^ T cell responses (antagonists) [6]. Typically, the sequences of antagonist peptides are variations of known agonist peptides [7], for example a single amino acid change in the minimum epitope of an agonist. These peptides are referred to as altered peptide ligands (APLs). Although it is clear that peptide sequence is important in T cell activation and antagonism, the mechanism by which these antagonist peptides work is unclear. These APLs not only fail to activate virus-specific T cells, but could potentially mediate escape from T cell recognition by blocking T cell responses directed to native virus sequence [8-12]. Moreover, Kent and colleagues have proposed CD4^+^ T cell antagonism as a potential mechanism for vaccine failure [13].

There are many studies to date looking at various potential mechanisms of T cell antagonism, including but not limited to systems with T cells expressing dual TCRs where one TCR can antagonize the other (cross-antagonism) [14-20], with most studies supporting the delivery of a dominant negative signal by antagonist peptides. Other proposed antagonism mechanisms include competitive inhibition leading to a failure to induce TCR signaling and Ca^++^ influx [21], and differential or negative signaling resulting from conformational changes of the TCR induced by the antagonist ligand [22,23]. There is also a study showing that T cell antagonism by galectin-1 binding results in truncated TCR signaling and disrupted lipid raft formation at TCR contact sites [24]. Taken together, it is apparent the mechanism of TCR antagonism is likely to vary depending on the model system.

Our earlier studies determined the minimum size epitopes from five HIV Gag-specific CD4^+^ T cell clones [4,25]. One clone, AC-25, has a minimum epitope 16 amino acids in length, PEVIPMFSALSEGATP (PP16), at positions 167–182 in Gag. N-terminal truncation of one amino acid allows for partial activation of T cell clones and further truncation eliminates activation [4]. Furthermore, a peptide truncated by three C-terminal amino acids (PEVIPMFSALSEG, PG13) also is non-activating and acts as an antagonist peptide [26,27]. X-ray diffraction analysis revealed that the extension of the C-terminal end of the PP16 peptide outside the peptide/MHC binding groove allows interaction with the TCR that does not occur with the truncated peptide, PG13 [27]. The native full-length epitope (PP16) can be engineered to act as an antagonist by substituting proline for glycine at position 13, and modeling shows that this eliminates a hinge region at the glycine and prevents interaction of the C-terminal portion of the peptide with the TCR [26]. Finally, we previously demonstrated that the antagonist peptide-MHC can bind the TCR, but with lower avidity than agonist peptide-MHC [26]. Given that we know many of the extracellular events that are associated with T cell antagonism in this system, we next wanted to determine the intracellular signals potentially delivered by cells exposed to the antagonist peptide. One of the limitations of the PG13 antagonist model is that we have not examined its abundance in vivo relative to the PP16 agonist peptide. However, peptide length-dependent antagonism of T cell responses has been previously described [28,29], and in a mouse model endogenous processing and antagonist activity of shorter peptides has been demonstrated [28].

In this study we examined the transcriptome and signaling cascade induced by the truncated antagonist peptide PG13. Utilizing CD4^+^ T cell clones, we showed that stimulation with the agonist peptide, PP16, in the presence of the antagonist, PG13, prevented transcriptional up-regulation of a broad group of activating proteins while also up-regulating a smaller number of inhibitory gene products. The majority of the down-regulated genes are associated with TCR signaling and pro-inflammatory cytokine production. We also observed truncated STAT phosphorylation and reduced production of multiple inflammatory cytokines and chemokines. We conclude that the truncated peptide acted as an antagonist to full length peptide through down-regulation of the T cell activation pathway, ultimately leading to global suppression of T cell signaling and function.

## Results

### Truncated STAT signaling after antagonist exposure

**Figure 1:**
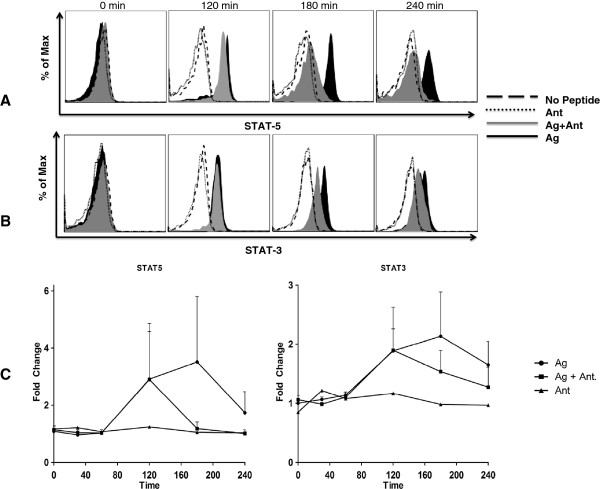
**Phosflow detection of STAT-3 and STAT-5.** STAT-3 and -5 were measured by flow cytometry in CD4^+^ T cell clones co-incubated with peptide (dotted = Ant, black = Ag, grey = Ag + Ant) or mock (dashed = no peptide) pulsed B cells. Panels **A** and **B** are one representative experiment of five. Bottom panels **(C)** show fold change in mean fluorescence intensity (MFI) of STAT-3 and -5 from five experiments; circle = Ag, square = Ag + Ant, triangle = Ant, error bars show standard error of the mean (SEM).

To understand the initial activation pathways disrupted in the presence of the antagonist peptide, the phosphorylation state of key molecules in the T cell activation pathway was measured [30,31]. Intracellular staining with antibodies specific for phosphorylated forms of proteins in the activation cascade allows longitudinal analysis of T cells after antigenic stimulation. We compared unstimulated T cell clones with clones stimulated with B cells pulsed with agonist (Ag) or antagonist (Ant) alone or agonist plus antagonist (Ag + Ant). Both Ag and Ag + Ant conditions induced comparable degrees of STAT-3 and STAT-5 phosphorylation two hours after stimulation, but phosphorylation was sustained at high levels only after Ag alone stimulation (Figure 1). These data suggest that antagonists do not completely block T cell activation, but rather truncate or attenuate the activation signal prior to full T cell activation.

### Inflammatory cytokines and chemokines are broadly down-regulated by antagonist exposure

**Figure 2:**
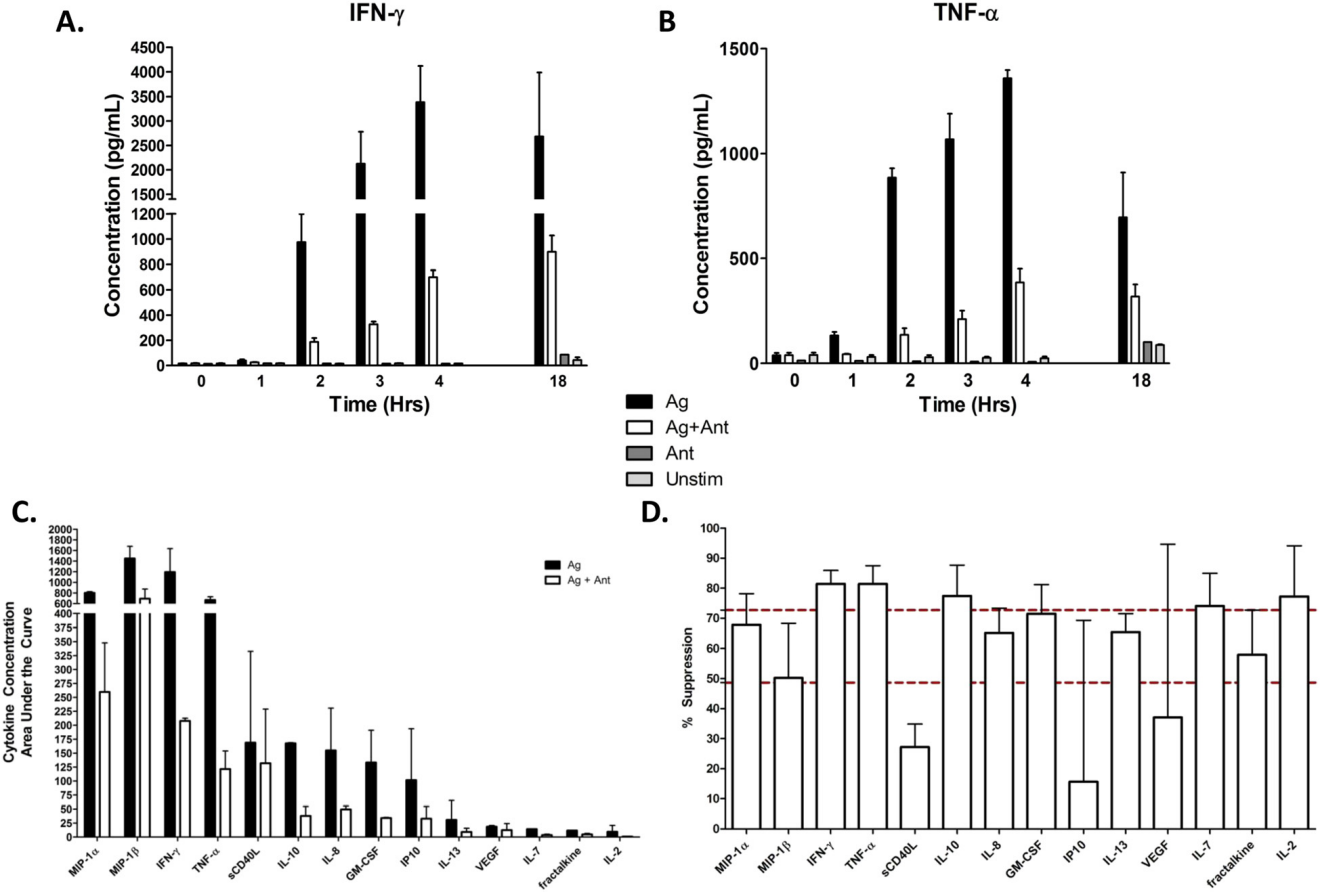
**Multiplex detection of secreted cytokines.** Secreted cytokines and chemokines following co-culture of CD4^+^ T cells with peptide or mock pulsed B cells were detected by multiplex assay. **(A)** and **(B)** Cytokine levels were tested in CD4^+^ T cell supernatants at serial time points over 18 hours with data for IFN-γ and TNF-α shown for two experiments (Ant treatment was run once). **(C)** Total area under the curve for secretion of each cytokine over 4 hours post-stimulation was calculated as described in Materials and Methods. Mean values from two replicate experiments are shown; error bars show SEM. **(D)** Percent suppression of agonist-induced cytokine suppression by addition of antagonist peptide. Suppression was calculated based on the AUC of cytokines four hours after stimulation. Dashed lines indicated the 95% confidence interval of the mean of 2 experiments, error bars show SEM.

Because the STAT-3 and STAT-5 phosphorylation data indicated incomplete activation of the T cells, we sought to determine what other T cell factors were decreased after Ag + Ant stimulation. Unstimulated T cells were compared to T cells stimulated with peptide pulsed B cells in the presence or absence of the antagonist peptide over an 18-hour time course (Figure 2A, B). At each time point, supernatants were harvested and measured for 39 secreted cytokines and chemokines by multiplex testing. Total area under the curve was determined for each of the cytokines secreted for the first four hours post-stimulation (see Methods). Cytokine/chemokine concentrations fell into three clusters, AUC > 500, AUC > 100, and AUC > 10 (Figure 2C). The highest expressed group (AUC > 500) was comprised of pro-inflammatory cytokines typically associated with T cell activation. As expected, there was a substantial decrease in IFN-γ production (5.7-fold) in the Ag + Ant treated samples. In addition, there were substantial decreases in several inflammatory cytokines, including MIP-1α (3.1-fold) and TNF-α (5.5-fold). The second group (AUC > 100) consisted mostly of growth and chemotactic factors, and although not expressed to the same degree as the pro-inflammatory cytokines, there were substantial decreases in expression in the Ag + Ant samples including GM-CSF (3.9-fold), IL-8 (3.1-fold), and IP-10 (3.1-fold). Also of note, a sizeable decrease in the regulatory cytokine IL-10 was detected (3.1-fold). The third group (AUC > 10) included chemotactic factors and the common γ-chain cytokines IL-2 and IL-7. Despite the low level secretion of these factors, several noteworthy decreases in expression were detected in the Ag + Ant samples, including IL-13 (3.4-fold), IL-7 (3.9-fold) and fractalkine (2.4-fold). We questioned whether T cell antagonism would selectively block pathways of activation (e.g. bias towards a Th2 response) and found that IFN-γ, TNF-α, and IL-10 were more suppressed and sCD40L was less suppressed than the rest of the cytokines measured in the Ag + Ant samples (Figure 2D). The degree of suppression ranged within the 95% confidence interval of 49% to 73% suppression for most of the cytokines, and it did not appear that the antagonist peptide selectively blocked a specific program of cytokine secretion, consistent with global suppression of T cell function by the antagonist.

### Antagonism causes global suppression of T cell activation

**Figure 3:**
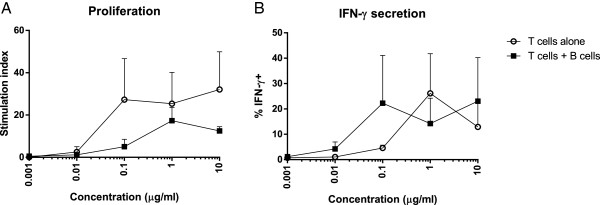
**T cell clones can present antigen in the absence of professional antigen presenting cells.** T cells were stimulated with Ag peptide in conditions with T cells alone or T cells and B cells mixed together in culture. Addition of peptide to T cell cultures lacking B cells still induced **(A)** proliferation and **(B)** IFN-γ secretion in the responding T cells at levels not significantly different from T cell cultures containing B cells (GEE p value >0.05). Proliferation was measured by ^3^H incorporation and the results are expressed as a stimulation index (counts in stimulated condition/counts in unstimulated condition). IFN-γ secretion was measured by intracellular staining for IFN-γ expression after stimulation in the presence of brefeldin A. Data are compiled from five proliferation experiments and three intracellular cytokine staining experiments. Error bars signify SEM.

**Figure 4:**
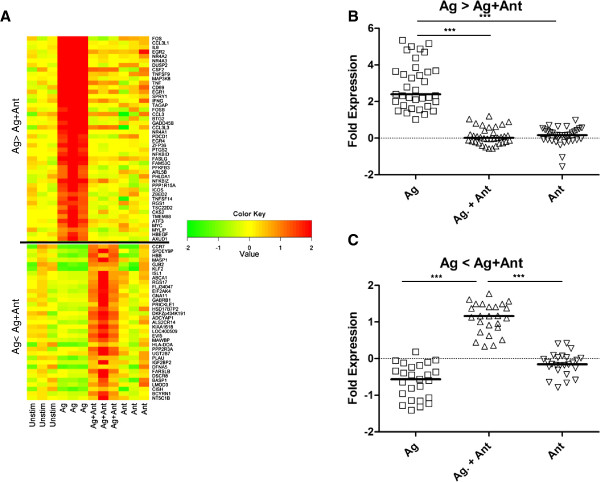
**Genes with 1.5 fold or greater difference in expression between Ag and Ag + Ant.** Fold change was calculated and Ag + Ant was subtracted from Ag treatment. Data were then sorted from highest to lowest and 1.5 fold or greater differences. Replicate values for up-regulated and down-regulated genes are displayed in a heat map **(A)**. Expression of up-regulated **(B)** and down-regulated **(C)** genes with medians from individual treatments are shown (Unstim = Unstimulated, Ag = Agonist, Ag + Ant = Agonist + Antagonist, Ant = Antagonist alone). p-Values were determined by Kruskal-Wallis followed by Dunn’s Multiple Comparisons Test, *** = p < 0.001.

To complement the functional studies, we surveyed expression of a broad array of over 46,000 genes after stimulation with the Ag, Ant, or Ag + Ant peptides. To maximize the signal derived from T cells, it would be optimal to exclude B cells from the gene array expression assay. The phenomenon of T cells acting as their own antigen presenting cells has been described previously [32], and we found that the AC-25 clones could present antigen to themselves (Figure 3). Given that the levels of stimulated T cells were not significantly different in the absence of B cells, B cells were excluded from the gene array experiments. We compared unstimulated T cells with T cells stimulated by Ag (PG16) in the presence or absence of Ant (PG13) for four hours, followed by mRNA extraction. Extracted mRNA was hybridized to an Illumina BeadArray, and the results were analyzed using IPA software. Genes with expression levels that differed by 1.5-fold or more with a p-value of <0.05 clustered into two distinct expression patterns, genes that were up-regulated in the Ag treatment and genes that were down-regulated in the Ag + Ant treatment (Figure 4A). Thirty five genes increased expression with Ag treatment, and their median expression was significantly higher than both Ag + Ant treatment as well as Ant alone treatment (Figure 4B). Conversely, a second set of 25 genes down-regulated in the Ag compared to the Ag + Ant treatment had significantly higher expression in the Ag + Ant treatment (Figure 4C). These data indicate that not only does the Ag + Ant treatment block up-regulation of genes triggered by the Ag, but Ag + Ant also up-regulates a subset of genes specific to its own genetic signature.

Table 1 shows the genes with 2-fold or greater difference in expression between the Ag and Ag + Ant treatment. Interestingly, most of the genes indicated are involved in T cell receptor signaling, including FOS/FOSB, EGR1/2, MAP3K8, and TAGAP, as well as in pro-inflammatory cytokine production, including TNFSF9, CCL3l1/3, IL-8, IFN-γ, and TNF. All of the genes expressed at higher levels in Ag compared to Ag + Ant treatment showed near zero levels of expression in the unstimulated cells, and weak to no induction in Ag + Ant or Ant stimulated cells. Of the few genes that had decreased expression in the Ag compared to Ag + Ant condition, most notably CCR7 and SPDEY9P, expression in the Ag condition was decreased compared to variable baseline expression and was modestly elevated in the Ag + Ant condition but not the Ant alone condition. Interestingly, SPDEY9P is a negative regulator of cell cycle progression [33]. These results depict a broad suppression of TCR signaling and T cell activation, consistent with our previously published results showing suppression of multiple arms of the immune response [26].

**Table 1:** Genes differing by 2-fold or more expression between Ag and Ag + Ant treatment

**Gene**	**Fold change**	**Function**	**Reference**
FOS	5.58	Immediate early gene transcription factor, up-regulated in response to extracellular signals like chemokines and cytokines	
CCL3L1	5.07	Codes for protein (Mip-1α) that binds CCR5 and caused internalization of CCR5, preventing HIV entry	[34,35]
IL8	4.81	Inflammatory cytokine produced by T cells, cell chemoattractant	
EGR2	4.75	Early growth response protein, responsible for regulation cell activation, overexpression leads to inhibition of T cell activation	[36]
NR4A2	4.62	Involved in T cell homeostasis	
NR4A3	4.33	Involved in T cell homeostasis	
DUSP2	3.83	Phosphatase that inactivate MAP kinase cascade, negatively regulate proliferation and differentiation	[37,38]
CSF2	3.49	Cell growth factor	
TNFSF9	3.26	TNF superfamily trans-membrane glycoprotein expressed on activated T cells	
MAP3K8	2.97	Activates MAP and JNK kinase pathways and induces NF-κB, promotes TNF-α and IL-2 during T cell activation	[39,40]
TNF	2.96	Pro-inflammatory cytokine	
CD69	2.94	Early inducible surface glycoprotein, functions as signal transducing receptor	
EGR1	2.85	Early growth response protein, enhances T cell function	[36]
SPRY1	2.77	Inhibits TCR signaling in differentiated cells, enhances TCR signaling in naïve cells	[41]
IFNG	2.71	Pro-inflammatory cytokine, antiviral and immunoregulatory functions	
TAGAP	2.63	Rho GTPase activating protein, role in T cell activation	
FOSB	2.62	Proteins that dimerize with JUN, form AP-1 transcription complex, role in proliferation and differentiation	[42]
CCL3	2.48	Pro-inflammatory cytokine (MIP-1α), natural ligand for CCR5	[43]
BTG2	2.47	Negative regulator of cell cycle	[44]
GADD45B	2.44	Increased levels observed following cell stress, inhibit cell growth	[45]
CCL3L3	2.44	Cytokine involved in immunoregulatory and inflammatory process, can bind to CCR5, inhibits HIV entry	[34,35]
NR4A1	2.27	Can block activation through NF-κB,	[46]
PDCD1	2.23	Negatively regulates TCR signals	[47]
EGR4	2.17	Interacts with NF-κB, controls transcription of genes encoding inflammatory cytokines including IL-2	[48]
ZFP36	2.1	Binds to 3’-UTR of some cytokines and promotes their degradation	[49]
PTGS2	2.09	Cox-2 has been shown to be Involved in Th1 differentiation	
NFKBID	2.03	Member of NF-κB inhibitor family	
FASLG	2.02	FAS-FASL binding is involved in T-cell homeostasis	
FAM53C	2.00	Unknown function	
GJB2	-2.13	Gap junction beta-2 protein	
HBB	-2.20	Haemoglobin beta subunit	
SPDEY9P	-2.25	Speedy/RINGO cell cycle regulator family member E9	
CCR7	-2.28	G-protein coupled family receptor involved in T cell migration	

Top up-regulated genes in gene array data.

Fold change was calculated and Ag + Ant was subtracted from Ag treatment. Data were then sorted from highest to lowest. The top genes that changed from Ag compared to Ag + Ant treatment are shown. Data are presented in Log base 2.

### Pathway analysis of microarray data

**Figure 5:**
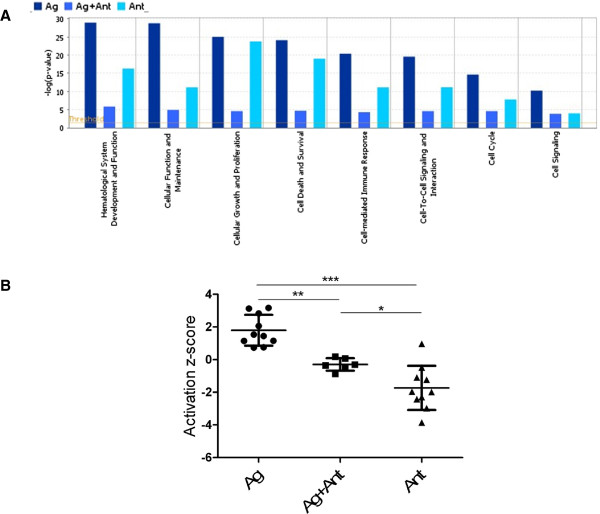
**IPA Comparison analysis of gene array data.** IPA comparison analysis was completed comparing Ag to Ag + Ant to Ant treatments. Displayed are molecular and cellular functions and physiological system development functions that are associated with cellular signaling and function and proliferation **(A)**. Each pair of bars displays the most significantly associated function within that category that is over-represented in the dataset. The larger the bar, the more strongly over-represented those functions were in the dataset. The p-values were determined by right-tailed Fisher’s exact test with a p < 0.05 considered significant. Only over-represented functions or ones that have more associated molecules than those are expected by chance are significant. Activation z-scores (calculated as described) for the 10 functions listed in Figure 6 that were associated with T cell signaling and function are displayed **(B)**. The p-values were determined by One-way analysis of variance followed by Tukey’s multiple comparison test. *p < 0.05, **p < .01, ***p < .005.

**Figure 6:**
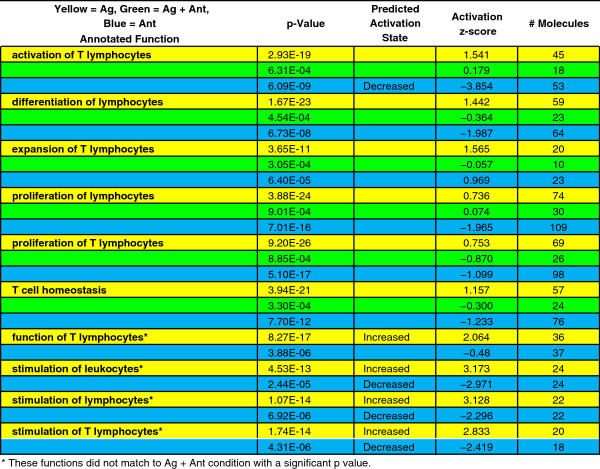
**Annotated biological functions associated with Hematological System Development and Function.** Annotated functions in the Hematological System Development and Function category that are associated with T lymphocytes with the number of molecules associated with those functions are indicated. Activation z-score as determined based on expression data following core analysis with an expression value cutoff of 0.5. Predicted activation state with activation z-score >2.0 or < -2.0. Yellow = Ag + Ant treatment, Green = Ag treatment.

We compared the pathway and functional enrichment results of the Ag and Ag + Ant treatment and observed significant differences in biological functions related to immune cell functions. The functions that scored most significantly in relation to each set of differentially expressed genes modulated by the indicated conditions are shown (Figure 5). For example, the function “activation of T lymphocytes”, which belongs to the broader category of Hematological System Development and Function, had a significant p-value of 2.39×10^-19^ for Ag treatment, whereas that same function had a p-value of 6.31×10^-4^ in the Ag + Ant treatment. Similar differences were seen in all of the functional categories shown in Figure 5 between Ag and Ag + Ant treatments. The larger the bar, the more strongly over-represented those functions were in the dataset, either up-regulated by the Ag or down-regulated by the Ant. Therefore, multiple categories that are related to cellular function and signaling were more strongly over-represented after Ag treatment compared to after Ag + Ant treatment of the clones. Furthermore, as shown in Figure 6, four functions (Function of T lymphocytes, Stimulation of Leukocytes, Lymphocytes, and T Lymphocytes) are predicted to be activated with the Ag treatment with z-score >2, whereas there was not a significant association between these functions and Ag + Ant treatment. With Ant treatment all but one function were decreased following Ant treatment, with four functions predicted to have decreased activation with z-score < -2. Activation of functional pathways was calculated as a z-score (see Methods), with a z-score >2 or < -2 considered significant [50]. Examining all the activation z-scores from Figure 6 revealed that activation was significantly higher after Ag stimulation than after Ag + Ant or Ant treatments (Figure 5B). Of note, activation z-scores were suppressed in the Ant condition compared to the Ag + Ant condition, consistent with the antagonist delivering a negative signal independently of the presence of the agonist peptide.

**Figure 7:**
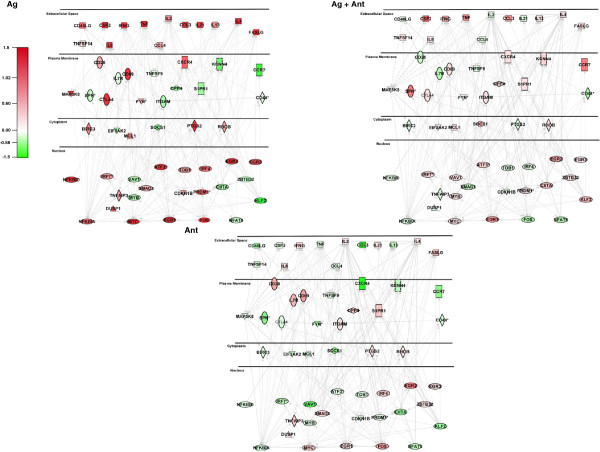
**Pathway analysis of gene array data.** We selected annotated functions within the Hematological system development and function category that were involved in all T cell functions and created a network from the 56 molecules represented in at least one of those functions. Molecules were connected based on direct (solid lines) and indirect (dotted lines) interactions and sorted based on subcellular localization. Expression values from Ag treatment, Ag + Ant treatment (Top Panels), and Ant treatment (Bottom Panel) were overlaid on the network, where red indicates positive fold changes and green represent negative fold changes. Square = Cytokine, Rectangle = G-protein coupled receptor, Dashed Rectangle = Ion Channel, Vertical Oval = Transmembrane Receptor, Horizontal Oval = Transcription regulator, Trapezoid = Transporter, Diamond = Enzyme, Hexagon = Peptidase, Pentagon = Phosphatase, Triangle = Kinase, Circle = Other, * = multiple expression value identifiers mapped to the same gene.

To further explore how the antagonist peptide influenced T cell function, annotated functions within the Hematological System Development and Function category were selected that were associated specifically with T lymphocytes. IPA defined functions included: proliferation of activated T cells, induction of T lymphocytes, stimulation of T lymphocytes, function of T lymphocytes, and activation of T lymphocytes. A network of direct and indirect interactions between the 56 molecules associated with at least one of these functions was built from the annotated functions, and then expression data from Ag, Ag + Ant, and Ant treatments was overlaid (Figure 7). When the expression data between the Ag and Ag + Ant treatments are contrasted, with the exception of several cytokine and plasma membrane receptors, it is apparent that the majority of the genes up-regulated by agonist were markedly reduced in expression when the antagonist peptide was present. Interestingly, there are several genes that were decreased in expression with Ag treatment which were increased in Ag + Ant treatment. These genes include C-C chemokine receptor 7 (CCR7), Kruppel Like Factor-2 (KFL-2), sphingosine-1 phosphate receptor-1 (S1PR1), the potassium channel KCCN, and suppressor of cytokine signaling-1 (SOCS-1). Of these, KFL-2 and SOCS-1 are known to be negative regulators of cellular activation [51,52]. Stimulation with Ant alone revealed further down-regulation of the genes in this cluster compared to the Ag + Ant treatment (Figure 7, bottom panel). Taken together, these data confirm that antagonism causes global suppression of gene associated cell signaling and activation along with induction of a smaller set of genes associated with reduced activation.

Microarray data were analyzed with IPA pathway analysis software with a log fold change cutoff of a half log, resulting in 481 and 383 molecules after Ag or Ag + Ant treatments, respectively. Following upload, we looked at the T cell receptor signaling canonical pathway with respect to our dataset. The IPA database has 109 genes annotated in the TCR signaling canonical pathway, 16 of which did not map to our dataset. However, of the remaining 93 mapped genes, there were 48 genes that were up-regulated and 45 genes down-regulated with Ag treatment. In contrast, for Ag + Ant treatment there were only 25 genes up-regulated and 68 genes that were down-regulated. Furthermore, when all gene expression changes were overlaid on the canonical pathway, the level of expression changes for genes that control TCR signaling could be compared for Ag (Additional file 1: Figure S1) and Ag + Ant (Additional file 1: Figure S2) conditions. There were considerable differences in the activation of the TCR signaling pathway. For example, several genes (C-FOS, CD28, IκBα, FYN) were highly up-regulated in the presence of the agonist but were down-regulated compared to unstimulated cells after Ag + Ant treatment. In addition to the genes that went from up-regulated to down-regulated, there were other genes, like CTLA4 and LAT, where the red color became less intense, indicating attenuated up-regulation after Ag + Ant stimulation. Stimulation with the Ant peptide resulted in further down-modulation of genes in the TCR signaling pathway compared to the Ag or Ag + Ant conditions (Additional file 1: Figure S3). Taken together, these observations represent decreased expression of a broad array of genes and indicate an overall suppression of TCR signaling when the antagonist is present.

**Figure 8:**
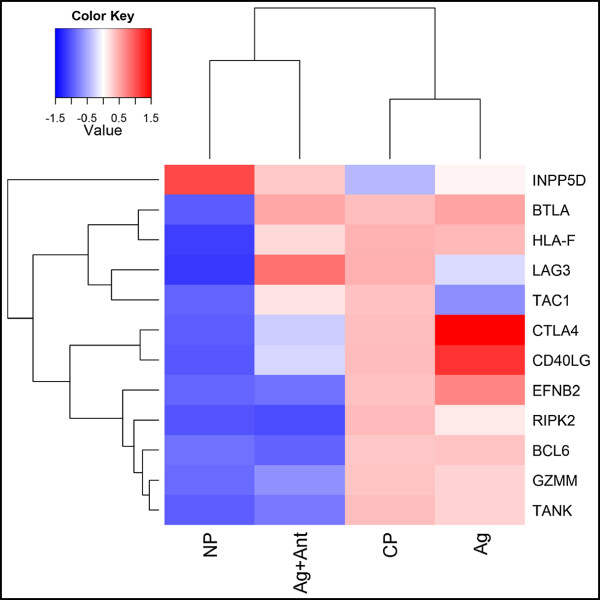
**Comparison with lymph node T cell responses of vaccinated macaques.** Twelve genes were found to be differentially expressed in non-protected vs. protected macaques, and z-scores for these genes in macaques and Ag and Ag + Ant stimulated T cells were used to generate a heat map. A z-score was calculated for each gene and then mapped by gene and treatment. For the macaque data the z-scores for 10 CP macaques and 4 NP macaques were averaged and mapped for comparison to Ag and Ag + Ant treatments. The clustering dendrogram was generated based on a hierarchical clustering algorithm with completed linkage and Euclidian distance. CP = completely protected macaque, NP = non-protected macaque.

In an independent study of T cell activation after SIV-LAV vaccine challenge in macaques [53], a specific pattern of gene expression representing T cell activation was observed in antigen-specific memory CD4^+^ T cells from the lymph nodes of protected macaques as early as day 4 post challenge, but not in non-protected macaques. Of note, the expression profile of the non-protected macaques is likely not driven by differences between the vaccine virus sequence and that of the challenge virus, as 2 of the 4 non-protected macaques and 8 of the 10 protected macaques received challenge with virus homologous in sequence to the vaccine virus [53]. We compared the expression pattern of these genes following Ag or Ag + Ant treatment to that of the protected (CP) and non-protected (NP) macaques (Figure 8). Of the 12 genes, 10 had the same expression trend in Ag vs. Ag + Ant as compared to CP vs. NP macaques. Concordant genes that are associated with T cell activation included CD40LG, CTLA4, BTLA, TANK [54-56]. Conversely, two genes LAG3 and TAC1 showed the opposite trends with activation in protected macaques and down-regulation after Ag stimulation, though only LAG3 is associated with T cell activation. Inclusion of data from Ant alone stimulated cells showed that gene expression was distinct from the other conditions, with Ant alone weakly grouping with the Ag and CP conditions (Additional file 2: Figure S4). These data indicate that the gene expression profile seen in T cell antagonism is similar to that of the memory CD4^+^ T cells from vaccinated but non-protected macaques.

## Discussion

In this study we shed new light on the activation of HIV-specific CD4^+^ T cell clones and the mechanism of T cell antagonism. We demonstrated that by four hours after stimulation, T cell activation was reduced at a global level in the presence of antagonist peptide. Phosphorylation occurred transiently in the presence of agonist plus antagonist peptides, but was attenuated and not sustained compared to agonist peptide alone stimulation. These data would be consistent with the antagonist peptide delivering a dominant negative signal to block T cell activation. Supporting this, the negative regulatory genes KFL-2, SOCS-1, and SPDEY9P were down-regulated after Ag stimulation and were in contrast up-regulated after Ag + Ant stimulation. Additionally, overall T cell activation pathways were down-regulated after stimulation with antagonist peptide, alone or in the presence of agonist peptide.

From prior work we know that antagonist peptide-MHC can bind to the TCR in the system studied here [27]. The initial induction of T cell signaling, including STAT-3 and -5 phosphorylation, at early time points with either Ag or Ag + Ant treatment supports delivery of an early activation signal. However, signaling is only sustained with the agonist alone condition, supporting generation of a dominant negative signal by the antagonist peptide (Figure 2). Although our data did not reach statistical significance due to the variability in overall intensity of the STAT signals, the differences observed were also confirmed by multiplex testing that measured the phosphorylation of STAT 3/5 (data not shown). STAT-3 and STAT-5 are both implicated in transducing signals delivered through the IL-2 receptor [30] and are phosphorylated in T cells as a result of IL-2 autocrine stimulation, so these data may reflect transient secretion of IL-2 after Ag + Ant stimulation. Additionally, STAT-3 and -5 are important for TCR antigen stimulation and are dependent on the MEK/ERK pathways [57]. Consistent with the STAT phosphorylation data, measurement of cytokines in culture supernatants showed a decrease in IL-2 after Ag + Ant compared to Ag stimulation.

A large number of the down-regulated genes in the Ag + Ant condition fell in the T cell signaling pathway, including FOS, FOSB, and MAP3K8, which are key parts in the MAPK and ERK pathways. These pathways lead to activation and dimerization of the FOS and Jun transcription factors, which form the AP-1 transcription factor complex [42], and subsequent regulation of differentiation and proliferation [58]. In addition to affecting these key pathway regulators, antagonism decreased transcription of genes coding for proteins responsible for T cell growth (EGR2), T cell homeostasis (NR4A2/3) and cell growth (CSF2). There were also several genes that were up-regulated only after Ag + Ant stimulation. Of these genes, CCR7, S1PR1, and KCCN are associated with T cell function and are induced by KLF-2 [59-61]. We observed increased KLF-2 expression, and it is possible that there was transient activation of Forkhead-Box transcription factor-1 (FOXO1A) in the Ag + Ant treatment that was not detected at four hours post-stimulation (data not shown), as KLF-2 expression is induced by FOXO1A [62,63]. Both FOXO1A and KLF-2 have been shown to modulate T cell activation and have been implicated in actively suppressing T cell function [51,62,64,65].

An important aspect of T cell activation is the production of cytokines and other molecules that mediate CD4^+^ T cell effector functions. We previously showed significant decreases in IFN-γ and serine esterase production [26]. These data confirmed reduced production of IFN-γ after Ag + Ant stimulation, and we also observed inhibition of several pro-inflammatory cytokines, including TNFα, MIP-1α/β, and IP-10, as well as chemotactic factors involved in the inflammatory response (GM-CSF and IL-8). In addition, the anti-inflammatory cytokine IL-10 was also suppressed after Ag + Ant stimulation. Prior studies have reported both increased [66,67] and decreased [68,69] IL-10 secretion after altered peptide ligand exposure. The mechanisms why some altered peptide ligands selectively induce IL-10 secretion while suppressing other T cell functions are not known but likely depend on the initial induction of the antigen-specific T cell and its plasticity at the time of stimulation.

The activation of SOCS1 in the Ag + Ant treatment is potentially important due to its known effect on cytokine signaling, as well as direct effects on T cell activation. SOCS1 has been shown to inhibit JAK tyrosine kinase activity [52], which is consistent with the significant reduction in STAT3/5 phosphorylation observed (Figure 2). Additionally, SOCS1 has been associated with modulation of T cell activation, where absence of SOCS1 leads to aberrant T cell activation [70,71]. Thus in Ag + Ant treatment, activation of SOCS1 could limit cytokine production as well as truncate T cell activation. Of similar importance is the increased expression of cell cycle regulating gene SPDEY9P. The RINGO/Speedy proteins are known to activate certain cyclin-dependent kinases, playing a critical role in cell-cycle progression and proliferation [72]. It was also found that SPDEY9P actually impairs cell cycle progression, acting as a negative regulator of cellular proliferation and cell cycle [44]. Thus, it is apparent that along with the down-regulation of multiple signaling pathways, Ag + Ant treatment also initiates a unique signaling cascade that activates negative regulators of cell cycle and proliferation including KFL-2 and SPDEY9P as well as cytokine production including SOCS1.

Interestingly, we saw a similar gene expression pattern in Ag vs. Ag + Ant as compared to CP vs. NP macaques in the previously published SIV-LAV vaccine study. In this study the early time point (4 days post challenge) observed in the CP vs. NP macaques, where viral load differences are not driving variation in the immune response, is reflective of what an effective antigen-specific immune response should look like, and T cell activation and NF-κB activity is important to this response. We showed above that following Ag + Ant treatment several members of the NF-κB signaling pathway were down-regulated, leading to the reduced expression of pro-inflammatory cytokines. In addition to this, in both NP macaques and Ag + Ant treatment we saw reduced expression of two key activators of NF-κB, TANK and RIPK2 [54,73]. We also saw similar reductions in CD40LG and the serine protease GZMM which both play a role in T cell activation and function of CD4 T cells. The reason for the similarity between the immune responses in non-protected macaques and Ag + Ant stimulated T cells is not clear. Since half the non-protected macaques received a challenge virus that matched the sequence of the vaccine [53], it is unlikely that sequence variation caused antagonism of T cells in the macaques. It is, however, notable that the effective vaccine response grouped with Ag stimulated T cells, and it would be of interest to determine if a T cell gene signature of an effective (or conversely ineffective) T cell response could be defined in studies of humans with good and poor control of HIV, as this would be a useful metric in assessing vaccine potency and potential for protection.

## Conclusions

In summary, this study demonstrated a broad reduction at the gene level of many factors associated with T cell activation, proliferation and function. In this system the antagonist peptide truncated activation induced by agonist peptide, consistent with delivery of a dominant negative signal. These data were corroborated with pathway analyses that demonstrated not only a global reduction in T cell receptor signaling, but also a global reduction in expression of a host of other genes that are involved in T cell proliferation, induction, activation, function and stimulation, along with the induction of a smaller set of genes implicated in suppressing T cell activation and function. We also provided a link to an existing vaccine study where Ag + Ant treatment elicited similar responses to those seen in vaccine failure. Thus, small changes to epitopes induced by HIV can have dramatic effects on CD4^+^ T cell function, highlighting an important obstacle to HIV vaccine development.

## Methods

### Study subject

Subject AC-25 was infected with HIV in 1998 and started highly active antiretroviral therapy within three months of seroconversion. The CD4^+^ T cell clone was derived from a sample obtained during a structured therapy intervention 18 months after infection. Samples were obtained with informed consent, and the studies were approved by the Massachusetts General Hospital Institutional Review Board.

### Peptides

The 16 amino acid agonist peptide PP16 (PEVIPMFSALSEGATP) and 13 amino acid antagonist peptide PG13 (PEVIPMFSALSEG) were obtained from Genemed Synthesis.

### Maintenance of CD4^+^ T cell clone and B cell line

The CD4^+^ T cell clone and autologous Epstein-Barr virus (EBV)-immortalized B cell line was derived as previously described [25]. The clones were cultured in RPMI media (UCSF Cell Culture Facility (CCF)) supplemented with penicillin/streptomycin (UCSF-CCF), HEPES (UCSF-CCF) and 20% fetal bovine serum (Hyclone) (R20) with 20x10^6^ gamma-irradiated peripheral blood mononuclear cells (PBMC) (90 Gy) and 50 IU/mL IL-2. Clones were re-stimulated every 2 weeks with fresh, irradiated feeder PBMC and 12 F6 (anti-CD3 antibody obtained from Johnson Wong, The Massachusetts General Hospital, 0.1 μg/mL) or purified anti-CD3 antibody (Clone UCHT-1, eBiosciences, 0.01 μg/mL). Autologous B cell lines were EBV transformed in the presence of cyclosporine A as previously described [25]. Cells were fed twice weekly with R20 and IL-2 (50 IU/ml).

### Antagonism assay

B cells were washed, counted and then pulsed for 1 hr with 0.1 μg/mL PP16. Cells were then washed and cultured 1:1 with T cell clones in the presence or absence of antagonist peptide (PG13) at 10 μg/ml for indicated times at 2x10^6^ cell/mL (250 μL total volume). For intracellular cytokine assays 10 μg/mL Brefeldin-A (Sigma Aldrich) was added after 1 hr of co-culture. Following co-culture, cells were washed and surface stained as indicated with Aqua Amine viability dye, CD3, CD4 and CD8, and then intracellularly stained for Phospho-Stat-3 and -5, or for IFN-γ. To ensure antagonism was present prior to further downstream analysis, 4 hr co-culture experiments to measure IFN-γ production were performed in parallel. For detection of secreted cytokines, co-cultures were incubated for indicated times without Brefeldin-A. Following incubation, cells were pelleted and supernatants were harvested and frozen at -80°C until analysis by multiplex assay. For detection of phosphorylated protein by multiplex assay, cells were co-cultured for indicated time points in 50 μL total volume in 96-well v-bottom dishes. Cells were washed with ice cold phosphate buffered saline (PBS) containing Halt phosphatase/protease inhibitor cocktail (Pierce), lysed with 50 μL Milliplex map lysis buffer (Millipore) and frozen at -80°C until analysis by multiplex assay. As CD4^+^ T cells were shown to be able to present antigen to themselves, in the microarray experiments the T cell clones were incubated with PP16 in the presence or absence of PG13 antagonist peptide without peptide-pulsed antigen presenting B cells to ensure that only T cell mRNA was extracted.

### Flow cytometry

Following co-culture cells were washed with PBS and pelleted. Cells were first labeled with Aqua Amine viability dye (Invitrogen) for 30 minutes and then subsequently labeled with CD3-PE, CD8-APC H7 (BD Biosciences), and CD4-BV421 (Biolegend) for 20 minutes. Cells were fixed and permeabilized with Caltag regents (Invitrogen). Staining for IFN-γ was performed using IFN-γ-APC (Biolegend) for 30 minutes. Cells were acquired on an LSR II flow cytometer (Becton Dickinson); 15,000 events per condition were collected.

### Phosflow

Following co-culture, cells were immediately fixed with BD Lyse/Fix buffer for 10 minutes (BD Biosciences), washed and then pelleted. Cells were then surfaced stained with CD4 Pacific Blue and CD8-APC H7 (BD Biosciences) for 20 minutes. For phosphorylated STAT-3 and -5 detection, samples were permeabilized with ice cold BD Perm Buffer III for 30 minutes, washed with PBS, and then incubated with phospho-STAT3 Alexa Fluor 488 or PE and phospho-STAT-5 Alexa Fluor 647 for 30 minutes. Cells were acquired on an LSR II flow cytometer (BD Biosciences); 50,000 events per condition were collected.

### Proliferation

To measure the ability of T cells to proliferate in response to peptide presented by B cells or T cells, proliferation was measured by ^3^H incorporation. T cell clones and B cells were each plated in triplicate wells at 50,000 cells/well in 96 well plates in R10. After 48 hours, 1 μCi of ^3^H-thymidine (Dupont Nen) in 50 μl R10 was added per well. Plates were harvested onto glass fiber filters after 18 hours and counts were obtained on a MicroBeta counter (Wallac). Results were expressed as stimulation index (SI), the ratio of counts from wells with antigen divided by the counts obtained from wells without antigen.

### Microarray

AC-25 clones were incubated with PP16 (without B cells) in either the presence or absence of antagonist peptide PG13 for 4 hours. Following incubation cells were flash frozen for microarray analysis. Total RNA was isolated using RNeasy Micro kits (Qiagen) and the quantity and quality confirmed using a NanoDrop 200 c (Thermo Fisher Scientific) and gel electrophoresis (Experion). Samples (50 ng) were amplified using Illumina TotalPrep RNA amplification kits (Ambion). Microarray analysis was conducted using 1.5 μg of biotinylated cRNA hybridized on Illumina Human WG-6 Expression Bead Chips (v2.0, Illumina) and then scanned on Illumina BeadStation 500GX. Raw data was collected with Illumina GenomeStudio software. Each hybridization was performed in triplicate. The data sets were pre-processed with quantile normalization, variance stabilization, and log_2_ transformation. Benjamini-Hochberg correction was employed to assess the occurrence of false positives by calculating the false discovery rate. Hierarchical clustering by Pearson’s correction and heatmap representations were generated using MultiExperiment Viewer v4.6.2 (Dana Farber Cancer Institute). Ingenuity Pathway Analysis v9 (IPA) (Ingenuity Systems) was used to select, annotate and visualize genes by function, network and canonical pathway.

### Multiplex cytokine assay

For detection of secreted cytokines, sample supernatants were assayed using the human cytokine/chemokine premixed 39-plex kit (Millipore) for epidermal growth factor (EGF), eotaxin, fibroblast growth factor (FGF)-2, Flt-3 ligand, fractalkine, G-CSF, GM-CSF, GRO, IFN-α2, IFN-γ, IL-10, IL-12 (p40), IL-12 (p70), IL-13, IL-15, IL-17, IL-1Rα, IL-1α, IL-1β, IL-2, IL-3, IL-4, IL-5, IL-6, IL-7, IL-8, IL-9, IP-10, monocyte chemotactic protein (MCP)-1, MCP-3, macrophage-derived chemokine (MDC), macrophage inflammatory protein (MIP)-1α, MIP-1β, TGF-α, TGF-β, vascular endothelial growth factor (VEGF), sCD40L, and sIL-2Rα following the manufacturer’s protocols. Standard curves and samples were run in duplicate. A Labscan 100 analyzer (Luminex) and Bio-Plex manager 4.1 (BioRad) were used to acquire samples. For detection of phosphorylated proteins, a Milliplex MAPmates kit (Millipore) was used for phospho-STAT-1 (Tyr701), phospho-STAT-3 (Tyr705), phospho-STAT-5A/B (Tyr694/Tyr699), phospho-STAT-6 (Tyr641), phospho-c-Jun (Ser73), phospho-AKT/PKB (Ser473), phospho-JNK/SAPK1 (Thr183/Tyr185), phospho-p38/SAPK1 (Thr180/Tyr182), phospho-p70 S6 kinase, and phospho-IkBα (Ser32) according to manufacturer’s protocols.

Area under the curve (AUC) at each time point was calculated for agonist (Ag) and agonist plus antagonist (Ag + Ant) treatment over a 240-minute period using concentration data from four equal 60 minute time intervals. The following variables and formula were used:

$$\begin{array}{ll} {\mathrm{Exp}}_{n} & =\;\mathrm{cytokine}\;\mathrm{concentration}\;\mathrm{at}\;\mathrm{time}\;\mathrm{point}\;n\;\mathrm{for}\;\mathrm{the}\;\mathrm{Ag}\;\mathrm{or}\;\mathrm{Ag}\\ & +\mathrm{Ant}\;\mathrm{experimental}\;\mathrm{condition} \end{array}$$

$$\begin{array}{ll} {\mathrm{Unstim}}_{n}= & \;\mathrm{cytokine}\;\mathrm{concentration}\;\mathrm{of}\;\mathrm{the}\;\mathrm{unstimulated}\;\\ & \mathrm{condition}\;\mathrm{at}\;\mathrm{time}\;\mathrm{point}\;n \end{array}$$

$${\mathrm{AUC}}_{n}=\;\mathrm{area}\;\mathrm{under}\;\mathrm{the}\;\mathrm{curve}\;\mathrm{from}\;\mathrm{time}\;\mathrm{point}\;n\;\mathrm{to}\;n+1$$

$$\begin{array}{ll} {\mathrm{AUC}}_{T}= & \mathrm{the}\;\mathrm{sum}\;\mathrm{of}\;\mathrm{the}\;\mathrm{AUC}\;\mathrm{for}\;\mathrm{each}\;\mathrm{of}\;\mathrm{the}\;\mathrm{four}\;\mathrm{time}\;\\ & \mathrm{intervals}\;\left(\mathrm{total}\;\mathrm{AUC}\right) \end{array}$$

$$\mathrm{AU}C_{n}=\;\;\left\{\left(\mathrm{Ex}p_{n}+\mathrm{Ex}p_{n+1}\right)\;-\;\left(\mathrm{Unsti}m_{n}+\mathrm{Unsti}m_{n+1}\right)\right\}/2$$

Percent suppression for each cytokine was calculated using the following:

$$\%\;\mathrm{Suppression}\;=\;\left(\left({\mathrm{AUC}}_{T}\mathrm{Ag}‒{\mathrm{AUC}}_{T}\mathrm{Ag}+\mathrm{Ant}\right)/{\mathrm{AUC}}_{T}\mathrm{Ag}\right)*100$$

### Ingenuity Pathway Analysis

BeadArray data were exported to Microsoft Excel and all required calculations [Ag – (Ag + Ant)] and organization were completed, and the resulting dataset was uploaded to IPA. Functional enrichment analysis was used to analyze the each treatment in the dataset. Genes were selected for pathway analysis using a log fold change cutoff of a half log (i.e. 1.4 fold change). This resulted in 431 and 383 analysis-ready molecules for Ag and Ag + Ant treatments respectively. We did a side-by-side comparison of the enrichment analysis results and analyzed the biological functions that were most over-represented in our datasets. The p-value associated with a specific biological process or function was calculated by IPA software and is a measure of the likelihood that molecules in the dataset that overlap with those functions do not occur by chance. The p-value was generated using a right-tailed Fisher’s exact test [74], and only over represented functions (ones that are significant) were displayed. The p-values displayed show the annotated function that has the most significant p-value for that category. The software also calculated a z-score, which represents a weighted sum based on evidence from the literature for the activating and inhibitory pattern of gene expression for a given function, to measure the net effects on broader biological functions, such as T cell proliferation. Up-regulation of genes associated with activation of a function (e.g. proliferation of T lymphocytes) or down-regulation of genes associated with inhibition of a function is predicted to be activating for that function, and the converse is true for inhibition of a function.

### Rhesus macaque gene expression data

The human gene expression data from the current work were compared to data obtained in a prior study of live attenuated SIV vaccines in rhesus macaques [53]. The details of the animals are included in the prior work, and the animals were used with approval of the Oregon National Primate Research Center’s Animal Care and Use Committee under the standards of the NIH Guide for the Care and Use of Laboratory Animals [53]. To normalize the gene expression data across the two species, Z-scores were calculated for each macaque or each treatment (e.g. Ag or Ag + Ant) on a gene by gene basis as follows:

$$\begin{array}{ll} \mathrm{score}= & \;\mathrm{gene}\;\mathrm{expression}\;\mathrm{value}\;\mathrm{for}\;\mathrm{each}\;\mathrm{individual}\\ & \;\mathrm{macaque}\;\mathrm{or}\;\mathrm{treatment} \end{array}$$

$$\begin{array}{ll} \mathrm{mean}= & \;\mathrm{average}\;\mathrm{gene}\;\mathrm{expression}\;\mathrm{of}\;\mathrm{all}\;\mathrm{macaques}\;\mathrm{or}\\ & \;\mathrm{treatments} \end{array}$$

$$\begin{array}{ll} \mathrm{standard}\;\mathrm{deviation}= & \;\mathrm{standard}\;\mathrm{deviation}\;\mathrm{of}\;\mathrm{the}\;\mathrm{mean}\\ & \;\mathrm{gene}\;\mathrm{expression}\;\mathrm{for}\;\mathrm{all}\;\mathrm{macaques}\\ & \;\mathrm{or}\;\mathrm{treatments} \end{array}$$

Z-scores for each gene were calculated using the following:

$$Z=\left(\mathrm{score}‒\mathrm{mean}\right)/\mathrm{standard}\;\mathrm{deviation}$$

### Statistical analysis

Comparison of the number of up-regulated and down-regulated genes across stimulation conditions was performed using a Kruskal-Wallis test followed by Dunn’s Multiple Comparison Test. Analysis of protein phosphorylation and cytokine data was performed using two-way analysis of variance (ANOVA) with Bonferroni’s correction for multiple comparisons. Comparison of T cell stimulation with and without B cells present was performed using generalized estimating equations (GEE). Statistical tests were performed using GraphPad Prism and R-Bioconductor software. p-value <0.05 was considered significant.

## Abbreviations

Ag: Agonist; Ag + Ant: Agonist + Antagonist; Ant: Antagonist; Unstim: Unstimulated; HAART: Highly active anti-retroviral therapy; STAT: Signal transducer and activator of transcription; AUC: Area under the curve; IPA: Ingenuity pathway analysis.

## Competing interests

The authors declare that they have no competing interests.

## Authors’ contributions

ESJ, DJK, and PJN conceived the study and designed the experiments. ESJ, JH, DP, AD, PJN, and LR performed the experiments. XD and MC performed statistical analysis. ESJ and PJN wrote the manuscript. All authors have read and approved the submission of the manuscript.

## Supplementary Material

Additional file 1: Figures S1–S3 T cell receptor signaling pathway analysis. Log-fold change was calculated from gene array mean fluorescence intensity data. Fold-change values were uploaded to Ingenuity Systems pathway analysis software. All expression values from the data set for genes associated with T cell receptor signaling were overlaid on the canonical pathway and compared. Fold-change values from Ag treatment (Left Panel) and Ag + Ant treatment (Right Panel) were overlaid on the T-cell receptor signaling canonical pathway. Red indicates positive fold-changes and green represents gene negative fold-changes. Molecules without an outline or color were not annotated in our dataset or fell below the expression threshold cutoff. The color scale was truncated at +/- 1.5 log-fold change. Click here for file

Additional file 2: Figure S4 Comparison with lymph node T cell responses of vaccinated macaques including Ant alone treatment. Twelve genes were found to be differentially expressed in non-protected vs. protected macaques, and z-scores for these genes in macaques and Ag, Ag + Ant, Ant stimulated T cells were used to generate a heat map. A z-score was calculated for each gene and then mapped by gene and treatment. For the macaque data the z-scores for 10 CP macaques and 4 NP macaques were averaged and mapped for comparison to T cell clone treatments. The clustering dendrogram was generated based on a hierarchical clustering algorithm with completed linkage and Euclidian distance. CP = completely protected macaque, NP = non-protected macaque. Click here for file
