# Supplementary figures and images for: A CD4+ T cell antagonist epitope down-regulates activating signaling proteins, up-regulates inhibitory signaling proteins and abrogates HIV-specific T cell function
Source: Retrovirology. 2014 Jul 5;11:57. doi: 10.1186/1742-4690-11-57 (PMC4227135; doi:10.1186/1742-4690-11-57)

## Slide 1
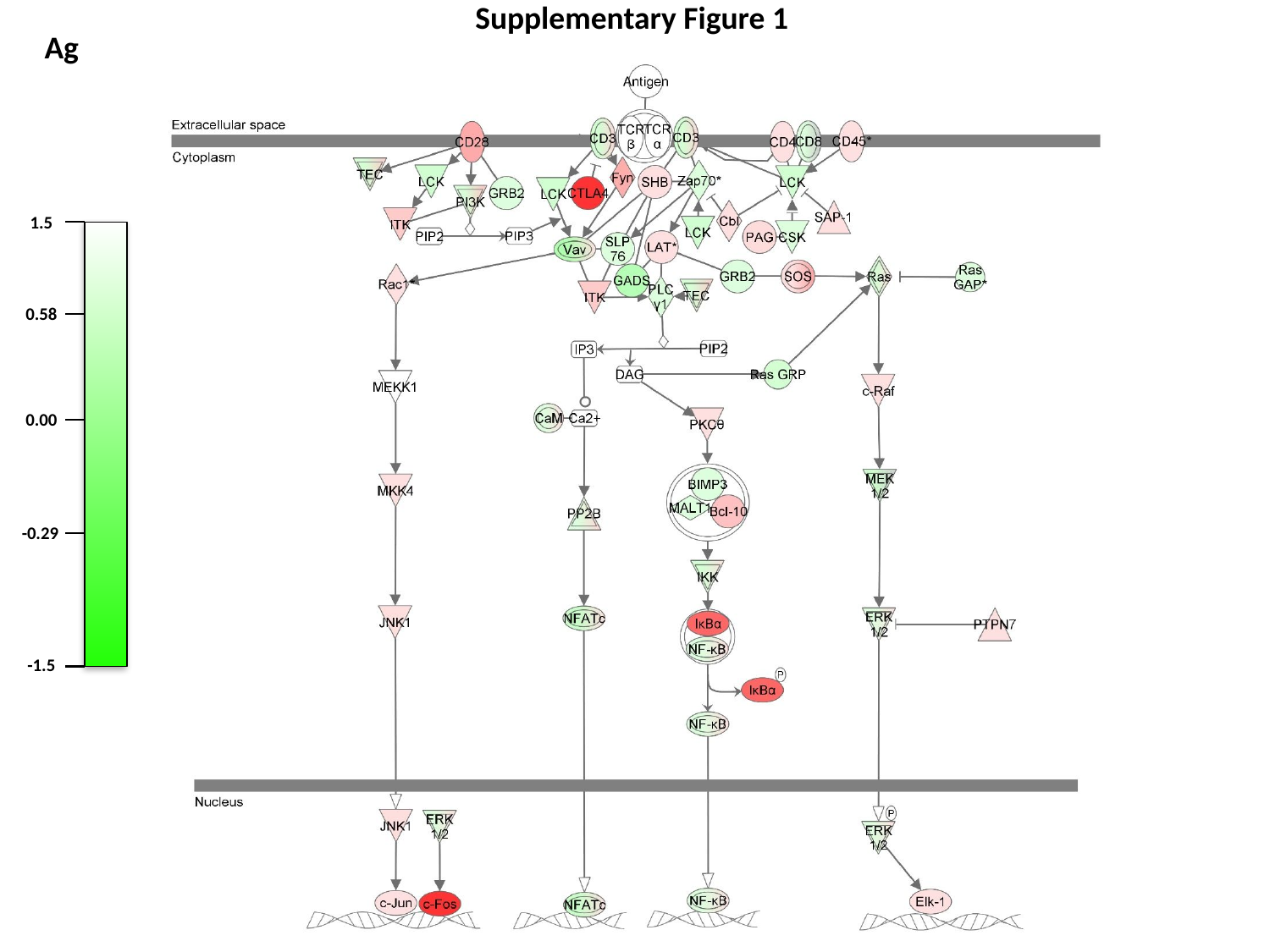

Supplementary Figure 1
Ag
1.5
0.58
0.00
-0.29
-1.5

Supplement: Additional file 1: Figures S1–S3 — T cell receptor signaling pathway analysis. Log-fold change was calculated from gene array mean fluorescence intensity data. Fold-change values were uploaded to Ingenuity Systems pathway analysis software. All expression values from the data set for genes associated with T cell receptor signaling were overlaid on the canonical pathway and compared. Fold-change values from Ag treatment (Left Panel) and Ag + Ant treatment (Right Panel) were overlaid on the T-cell receptor signaling canonical pathway. Red indicates positive fold-changes and green represents gene negative fold-changes. Molecules without an outline or color were not annotated in our dataset or fell below the expression threshold cutoff. The color scale was truncated at +/- 1.5 log-fold change. [file 1742-4690-11-57-S1.zip › 1043899278121280_add1/1043899278121280_add1.pptx]

## Slide 1
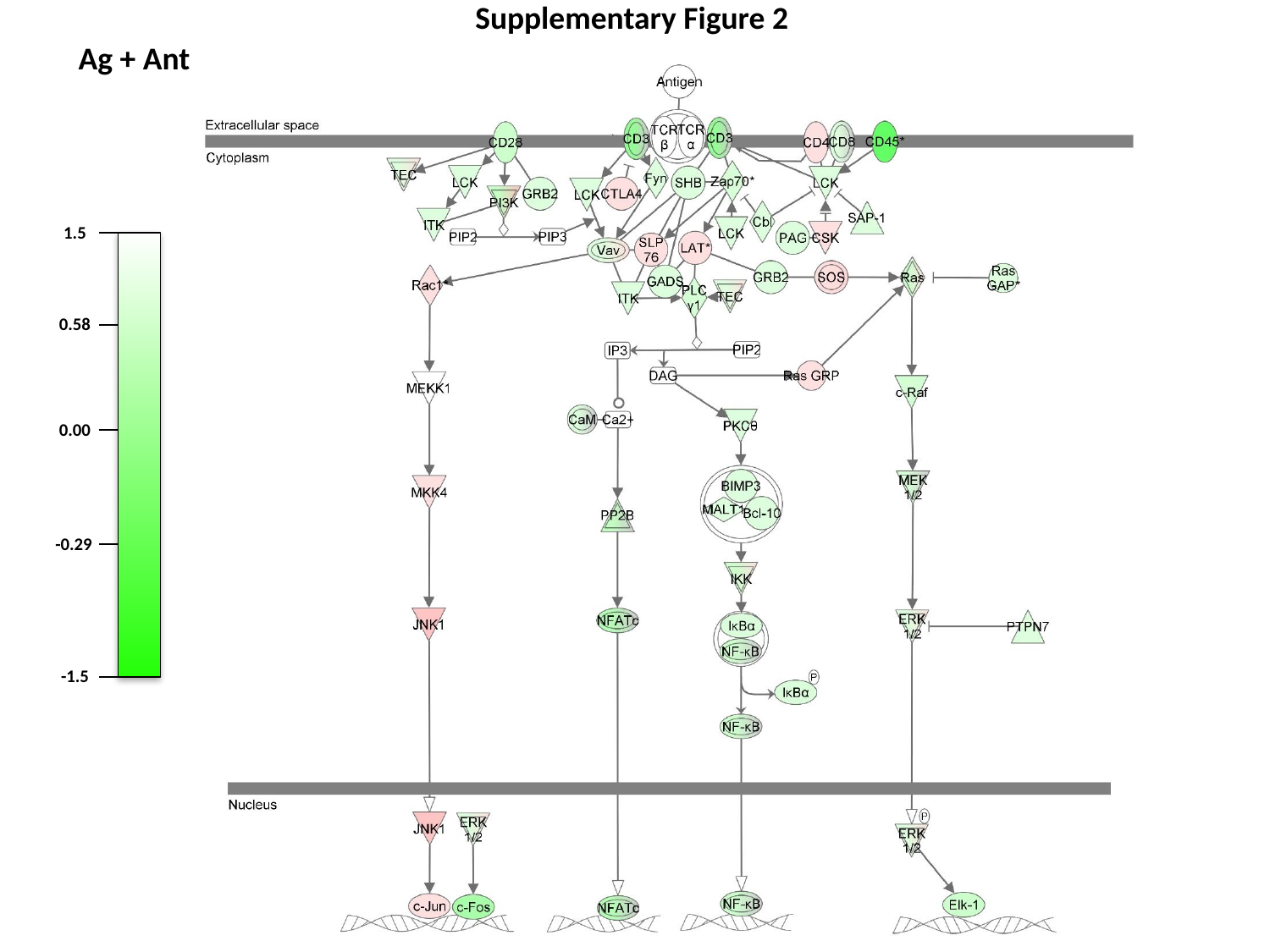

Supplementary Figure 2
Ag + Ant
1.5
0.58
0.00
-0.29
-1.5

Supplement: Additional file 1: Figures S1–S3 — T cell receptor signaling pathway analysis. Log-fold change was calculated from gene array mean fluorescence intensity data. Fold-change values were uploaded to Ingenuity Systems pathway analysis software. All expression values from the data set for genes associated with T cell receptor signaling were overlaid on the canonical pathway and compared. Fold-change values from Ag treatment (Left Panel) and Ag + Ant treatment (Right Panel) were overlaid on the T-cell receptor signaling canonical pathway. Red indicates positive fold-changes and green represents gene negative fold-changes. Molecules without an outline or color were not annotated in our dataset or fell below the expression threshold cutoff. The color scale was truncated at +/- 1.5 log-fold change. [file 1742-4690-11-57-S1.zip › 1043899278121280_add1/1043899278121280_add2.pptx]

## Slide 1
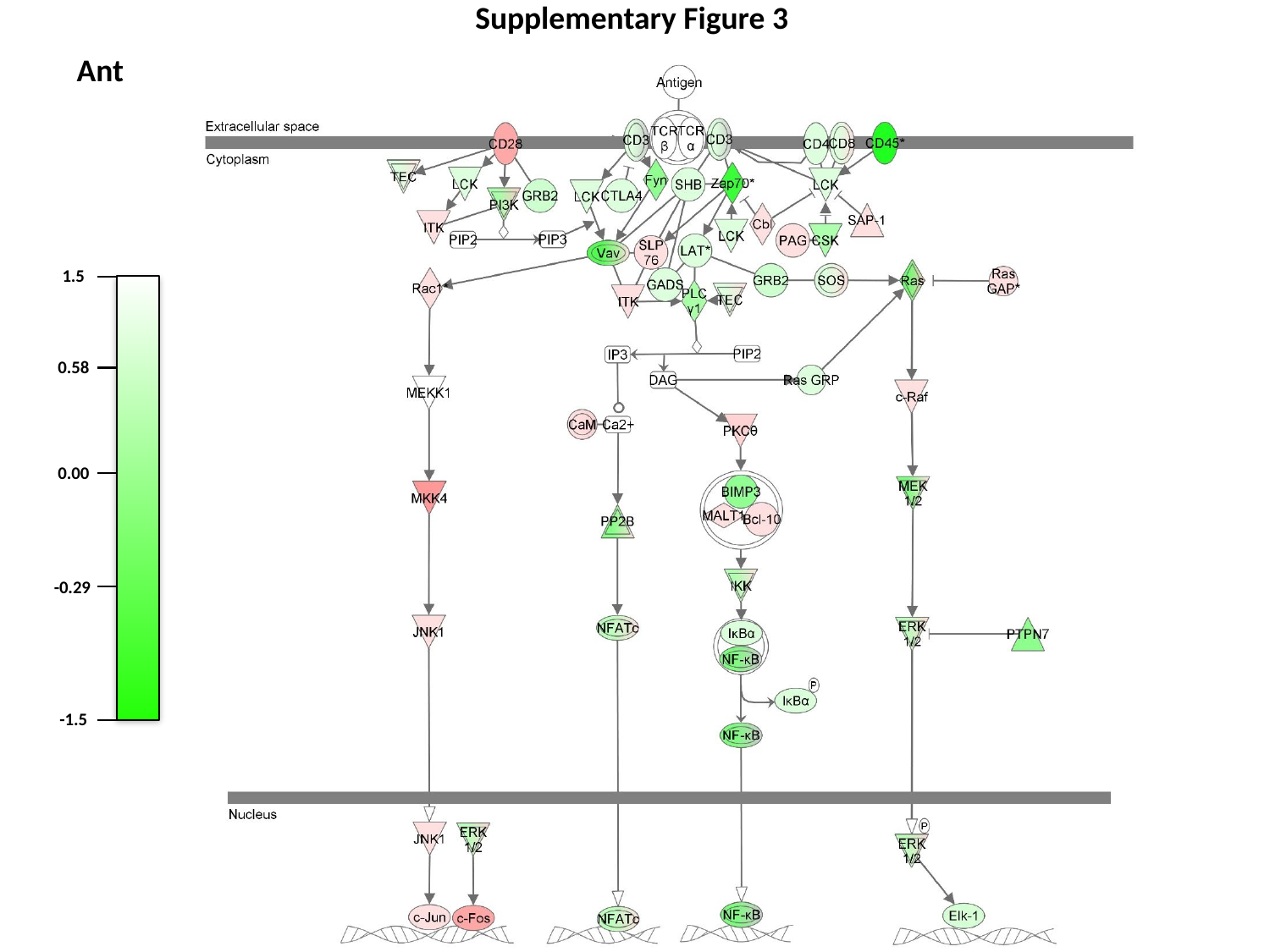

Supplementary Figure 3
Ant
1.5
0.58
0.00
-0.29
-1.5

Supplement: Additional file 1: Figures S1–S3 — T cell receptor signaling pathway analysis. Log-fold change was calculated from gene array mean fluorescence intensity data. Fold-change values were uploaded to Ingenuity Systems pathway analysis software. All expression values from the data set for genes associated with T cell receptor signaling were overlaid on the canonical pathway and compared. Fold-change values from Ag treatment (Left Panel) and Ag + Ant treatment (Right Panel) were overlaid on the T-cell receptor signaling canonical pathway. Red indicates positive fold-changes and green represents gene negative fold-changes. Molecules without an outline or color were not annotated in our dataset or fell below the expression threshold cutoff. The color scale was truncated at +/- 1.5 log-fold change. [file 1742-4690-11-57-S1.zip › 1043899278121280_add1/1043899278121280_add3.pptx]

## Slide 1
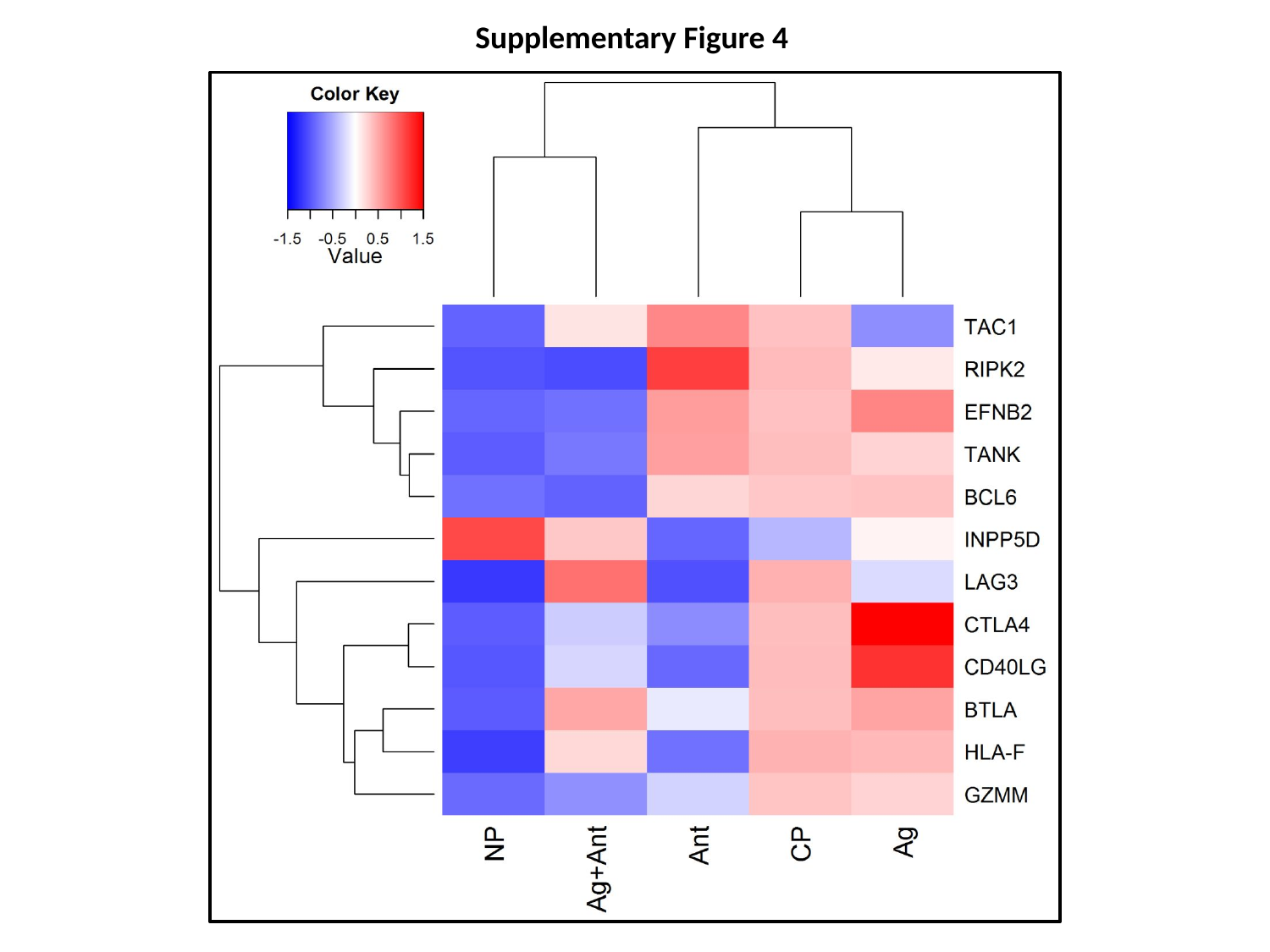

Supplementary Figure 4

Supplement: Additional file 2: Figure S4 — Comparison with lymph node T cell responses of vaccinated macaques including Ant alone treatment. Twelve genes were found to be differentially expressed in non-protected vs. protected macaques, and z-scores for these genes in macaques and Ag, Ag + Ant, Ant stimulated T cells were used to generate a heat map. A z-score was calculated for each gene and then mapped by gene and treatment. For the macaque data the z-scores for 10 CP macaques and 4 NP macaques were averaged and mapped for comparison to T cell clone treatments. The clustering dendrogram was generated based on a hierarchical clustering algorithm with completed linkage and Euclidian distance. CP = completely protected macaque, NP = non-protected macaque. [file 1742-4690-11-57-S2.zip › 1043899278121280_add2/1043899278121280_add4.pptx]

## Slide 1
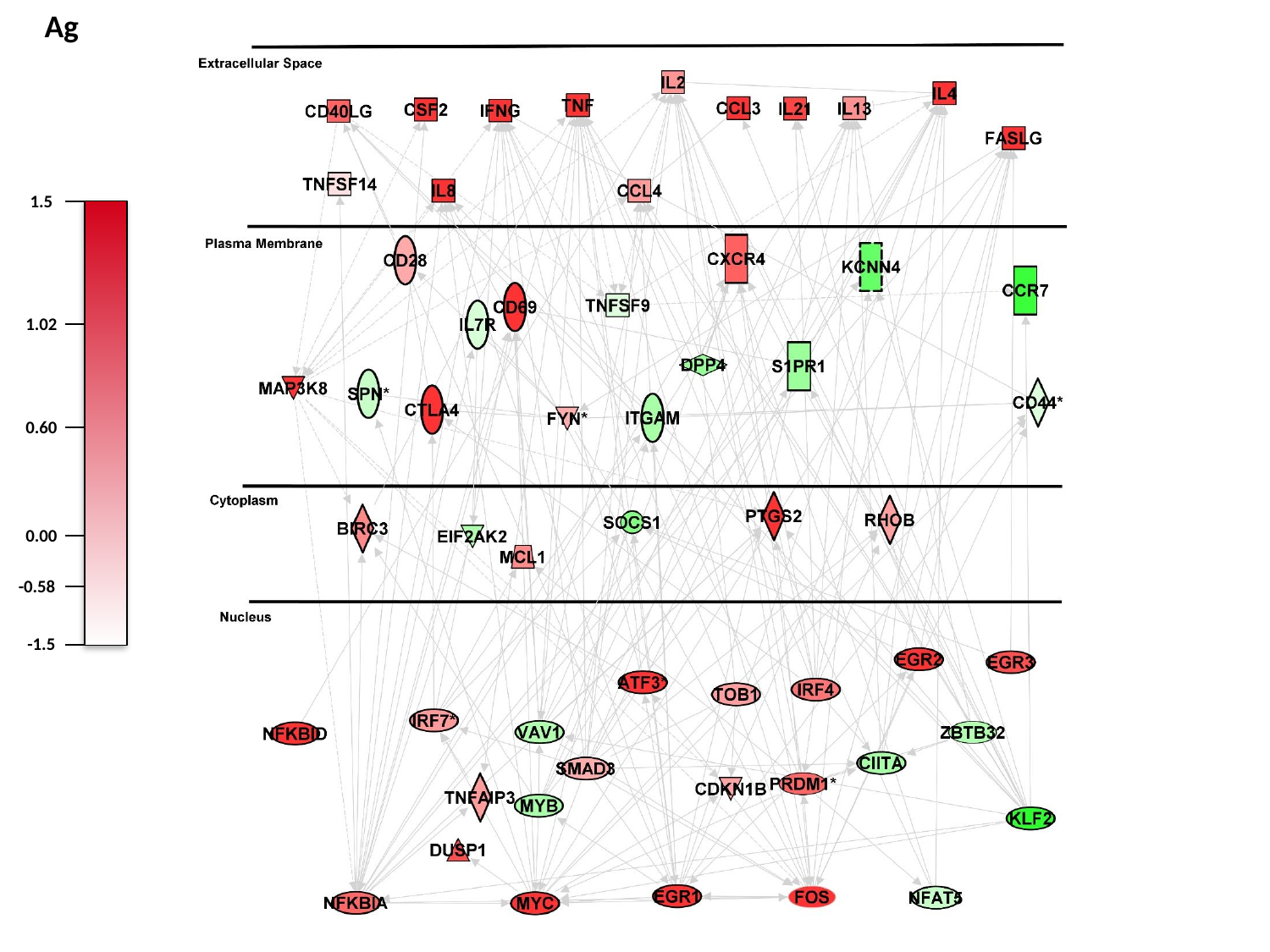

Ag
1.5
1.02
0.60
0.00
-0.58
-1.5

Supplement: Additional file 2: Figure S4 — Comparison with lymph node T cell responses of vaccinated macaques including Ant alone treatment. Twelve genes were found to be differentially expressed in non-protected vs. protected macaques, and z-scores for these genes in macaques and Ag, Ag + Ant, Ant stimulated T cells were used to generate a heat map. A z-score was calculated for each gene and then mapped by gene and treatment. For the macaque data the z-scores for 10 CP macaques and 4 NP macaques were averaged and mapped for comparison to T cell clone treatments. The clustering dendrogram was generated based on a hierarchical clustering algorithm with completed linkage and Euclidian distance. CP = completely protected macaque, NP = non-protected macaque. [file 1742-4690-11-57-S2.zip › 1043899278121280_add2/1043899278121280_add4Ag.pptx]

## Slide 1
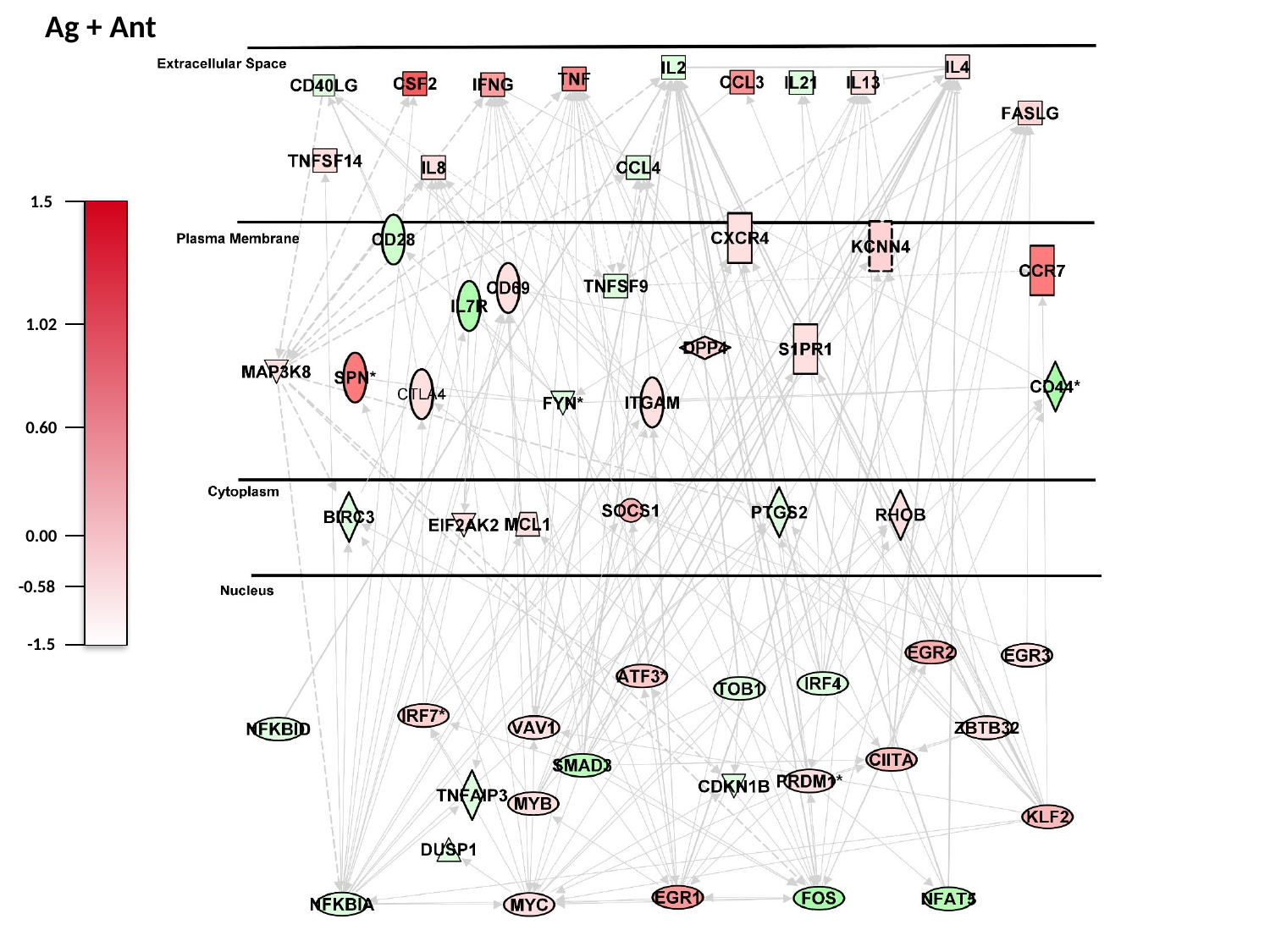

Ag + Ant
1.5
1.02
0.60
0.00
-0.58
-1.5

Supplement: Additional file 2: Figure S4 — Comparison with lymph node T cell responses of vaccinated macaques including Ant alone treatment. Twelve genes were found to be differentially expressed in non-protected vs. protected macaques, and z-scores for these genes in macaques and Ag, Ag + Ant, Ant stimulated T cells were used to generate a heat map. A z-score was calculated for each gene and then mapped by gene and treatment. For the macaque data the z-scores for 10 CP macaques and 4 NP macaques were averaged and mapped for comparison to T cell clone treatments. The clustering dendrogram was generated based on a hierarchical clustering algorithm with completed linkage and Euclidian distance. CP = completely protected macaque, NP = non-protected macaque. [file 1742-4690-11-57-S2.zip › 1043899278121280_add2/1043899278121280_add4Ag+ant.pptx]

## Slide 1
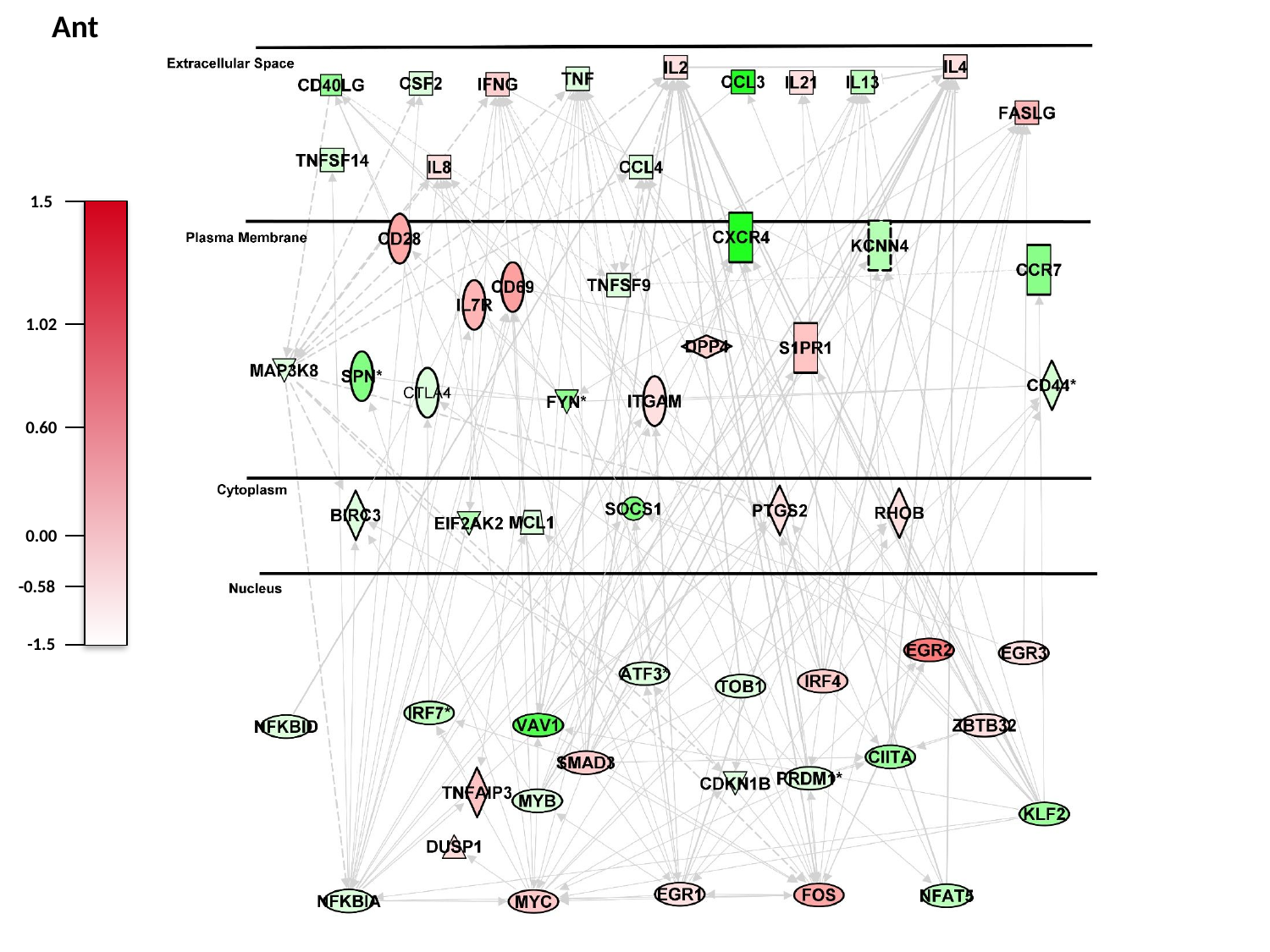

Ant
1.5
1.02
0.60
0.00
-0.58
-1.5

Supplement: Additional file 2: Figure S4 — Comparison with lymph node T cell responses of vaccinated macaques including Ant alone treatment. Twelve genes were found to be differentially expressed in non-protected vs. protected macaques, and z-scores for these genes in macaques and Ag, Ag + Ant, Ant stimulated T cells were used to generate a heat map. A z-score was calculated for each gene and then mapped by gene and treatment. For the macaque data the z-scores for 10 CP macaques and 4 NP macaques were averaged and mapped for comparison to T cell clone treatments. The clustering dendrogram was generated based on a hierarchical clustering algorithm with completed linkage and Euclidian distance. CP = completely protected macaque, NP = non-protected macaque. [file 1742-4690-11-57-S2.zip › 1043899278121280_add2/1043899278121280_add4Ant.pptx]
